# The effect of final irrigation with different solutions on smear layer removal and dentin erosion: A scanning electron microscope study

**DOI:** 10.1371/journal.pone.0308606

**Published:** 2024-08-09

**Authors:** Mohammed AlBatati, Ammar AbuMostafa

**Affiliations:** 1 Department of Endodontics, Ministry of Health, Dental Speciality Center in Althagr General Hospital, Jeddah, Saudi Arabia; 2 Department of Restorative Dentistry, College of Medicine and Dentistry, Riyadh Elm University, Riyadh, Saudi Arabia; Universidade Federal Fluminense, BRAZIL

## Abstract

**Aim:**

This study aimed to compare the effectiveness of initial irrigation with sodium hypochlorite (NaOCl) and final irrigation with QMix, 40% citric acid, and 17% ethylenediaminetetraacetic acid (EDTA) on smear layer removal and dentin erosion.

**Methodology:**

Forty extracted human mandibular premolar teeth were randomly divided into four groups (*n* = 10) according to the type of final irrigants used: 17% EDTA, QMix, citric acid, and control (normal saline). Canals were mechanically prepared using ProTaper Next instruments to an apical size of X3. Subsequently, the roots were sectioned in a buccolingual direction. Scanning electron microscopy (SEM) was used to assess the presence of the smear layer and the amount of dentin erosion in the coronal, middle, and apical thirds of the root canals.

**Results:**

In regards to smear layer removal, there was a significant difference between the control group and the other tested groups. Moreover, it was significantly higher in the coronal and middle thirds than in the apical third. However, there were no significant differences between the groups of EDTA, QMix, and citric acid. Concerning dentin erosion, citric acid produced significantly more dentin erosion than the other tested groups.

**Conclusion:**

Final irrigation with solutions had a higher ability to remove the smear layer in the coronal and middle thirds compared to the apical third. Of all the solutions tested, 40% citric acid had the most pronounced impact on dentin erosion, followed by 17% EDTA and QMix.

## 1. Introduction

The critical component of root canal treatment is biomechanical or chemomechanical preparation. Both mechanical debridement and the use of specific chemical irrigants are deemed crucial for successful root canal therapy. An optimal irrigation solution should have sufficient antibacterial efficacy and be capable of removing organic tissues, debris, and the smear layer from the root canal system [1].

The smear layer is a tenaciously adherent layer that forms on the canal walls and can reduce the permeability of dentin to canal medicaments and irrigants [2]. This layer has a thickness ranging from 1–2 μm. Under scanning electron microscopy (SEM), it appears amorphous and uneven. The smear layer is composed of inorganic and organic constituents. The organic constituents include bacterial debris and pulpal remnants, while the inorganic constituents are dentinal debris [2]. The prevailing recommendation is to eliminate this layer, as its retention can induce apical and coronal microleakage. Additionally, leaving the smear layer may hinder and weaken the bonding process of root canal sealant with dentinal walls, leading to a failure of the root canal treatment [3].

Sodium hypochlorite (NaOCl) is considered one of the most widely utilized irrigation solutions due to its antibacterial properties and potential to dissolve necrotic tissue [4, 5]. However, given its inadequacy in removing the inorganic components of the smear layer, it is advised that a chelating agent or an acid be used in conjunction with NaOCl for the final irrigation of root canals [3, 6]. Possible chemical changes in the root canal dentin structure during the demineralization phase using chelating agents have been documented to create erosive alterations and reduce dentin hardness [7, 8], which may adversely impact the sealing capacity and adherence of root canal sealers [9]. Insufficient adhesion of root canal filling material and the absence of a hermetic seal might result in microleakage and bacterial invasion, jeopardizing the outcome of root canal therapy.

The predominant strategy for effective disinfection, smear layer removal, and dissolving organic tissue in root canals is a final irrigation routine that involves the alternating utilization of NaOCl and ethylenediaminetetraacetic acid (EDTA). However, the use of EDTA during root canal treatment has numerous possible drawbacks.

Initially, it has been found that combining EDTA and NaOCl reduces the amount of freely available chlorine present in the combination, which decreases the antibacterial and tissue-dissolving abilities of NaOCl [10]. Furthermore, the efficacy of EDTA in removing the smear layer is reduced in the apical portion of the root canals, apparently due to the high surface tension, which decreases its wettability [11], and a decline in the amount of non-collagenous organic matrix in that area. Additionally, EDTA has a low capability to disinfect dentinal tubules [12]. As a result, different chelating regimens have been investigated in recent years to overcome the drawbacks of EDTA.

Citric acid is an organic acid that serves as a potent irrigation solution for eliminating the smear layer, with concentrations ranging from 10% to 50% [13]. Studies have shown that citric acid removes the smear layer more effectively than other solutions, such as EDTA, H_2_O_2_, and phosphoric acid [14].

To simultaneously achieve the objectives of smear layer removal and disinfection and simplify the irrigation technique, a combined chemical agent has been introduced as a final irrigant following NaOCl: QMix (Dentsply Tulsa Dental Specialties, Tulsa, OK, USA). QMix is composed of a chelating agent (17% EDTA), an antimicrobial agent (2% chlorhexidine [CHX]), and a detergent (cetrimide) [15]. Numerous studies have explored the comparative effectiveness in smear layer removal between QMix, citric acid, and 17% EDTA. However, the acquired data have not led to a conclusion regarding the most effective irrigant solution [7, 15–20]. To date, no studies have assessed the general effectiveness of QMix, 40% citric acid, and EDTA in eliminating the smear layer and their influence on dentin erosion. Furthermore, there are no published studies specifically comparing the efficacy of these agents as final irrigation solutions, especially when used in conjunction with NaOCl.

The aim of this in vitro study was to compare the effects of initial irrigation with NaOCl and final irrigation with QMix, 40% citric acid, and 17% EDTA on smear layer removal and dentin erosion. The null hypothesis posited that there would be no significant difference in the efficacy of these irrigation solutions concerning smear layer removal and dentin erosion. At the time of conducting this study, there was no previous published article comparing the effects of these irrigants together on smear layer removal and dentin erosion.

## 2. Materials and methods

### 2.1. Selection and preparation of the samples

Ethical approval for this study was obtained from the institutional review board (IRB) of Riyadh Elm University (REU) (No. FPGRP/2021/637/647/646). A total of 40 single-rooted human mandibular premolar teeth were extracted from patients for orthodontic purposes and stored in normal saline (Sidalih, Saudi Arabia) until further analysis to prevent dehydration. Teeth were sterilized using an autoclave at 121 C° at a pressure of 30 psi for 20 min (CISA 420, Italy) then accessed on January 3^rd^ 2023. Criteria for inclusion in the study were intact teeth, type I according to Vertucci’s classification, no previous endodontic treatment, no coronal restoration, no resorption, and a closed apex. Periapical X-rays (Dentsply Sirona, USA) of the selected teeth were captured to confirm the presence of a single root canal and a normal pulp chamber, as well as to ensure the absence of root caries, prior root canal treatment, resorptions, cracks, calcifications, and immature apices. The crowns of the teeth were sectioned to achieve a standard length of 15 mm using a diamond disc (Kerr, USA). An International Organization for Standardization (ISO) size #10 K-file (Dentsply Sirona, Ballaigues, Switzerland) was inserted into the root canals until the tip of the file was visible at the apical foramen, and the working length was determined by subtracting 1 mm from the recorded distance of the apical foramen. Before the chemomechanical treatment of the root canals, the teeth were randomly allocated into four groups (*n* = 10) based on the solution used for the final irrigation:

■ Group 1: Control (normal saline);
■ Group 2: QMix (Dentsply Tulsa Dental Specialties, Tulsa, OK, USA);
■ Group 3: 17% EDTA (MD-Cleanser, Meta Biomed, Chungbuk, Korea);
■ Group 4: 40% citric acid (Cerkamed, PPH Cerkamed, Stalowa Wola, Poland).

Subsequently, the root canals were mechanically prepared according to the manufacturer’s instructions concerning speed (300 rpm), torque (200 gcm), and technique using ProTaper Next rotary instruments (Dentsply Sirona, USA) to an apical size of X3 (30/0.07) that were coupled to an electric motor (X-Smart; Dentsply Maillefer, USA). During instrumentation and after the use of each instrument, the canals were irrigated with 5 ml of 5.25% NaOCl for 2 min.

### 2.2. Final rinse protocols

Table 1 presents the final irrigation protocols applied after instrumentation. After the final irrigation, the root canals were rinsed with normal saline to remove any precipitate and dried with sterile paper points that matched ProTaper Next file X3 for optimal results (Dentsply Sirona, USA). All irrigating solutions were introduced with a 30-G (diameter 0.3 mm) syringe needle (Fanta Dental Side Vented Tips, Fanta Dental Materials, China), which was inserted up to 1–2 mm from the working length.

**Table 1:** Final rinse protocols.

NaOCI				Normal Saline		Final irrigants		
Group	ml	Time (min)	Conc. (%)	ml	Time (min)	ml	Time (min)	Final irrigant
**Control**	5	2	5.25	5	2	5	2	Saline
**Qmix**	5	2	5.25	5	2	5	2	Qmix
**EDTA**	5	2	5.25	5	2	5	2	17% EDTA
**Citric Acid**	5	2	5.25	5	2	5	2	40% citric acid

**Fig 1:**
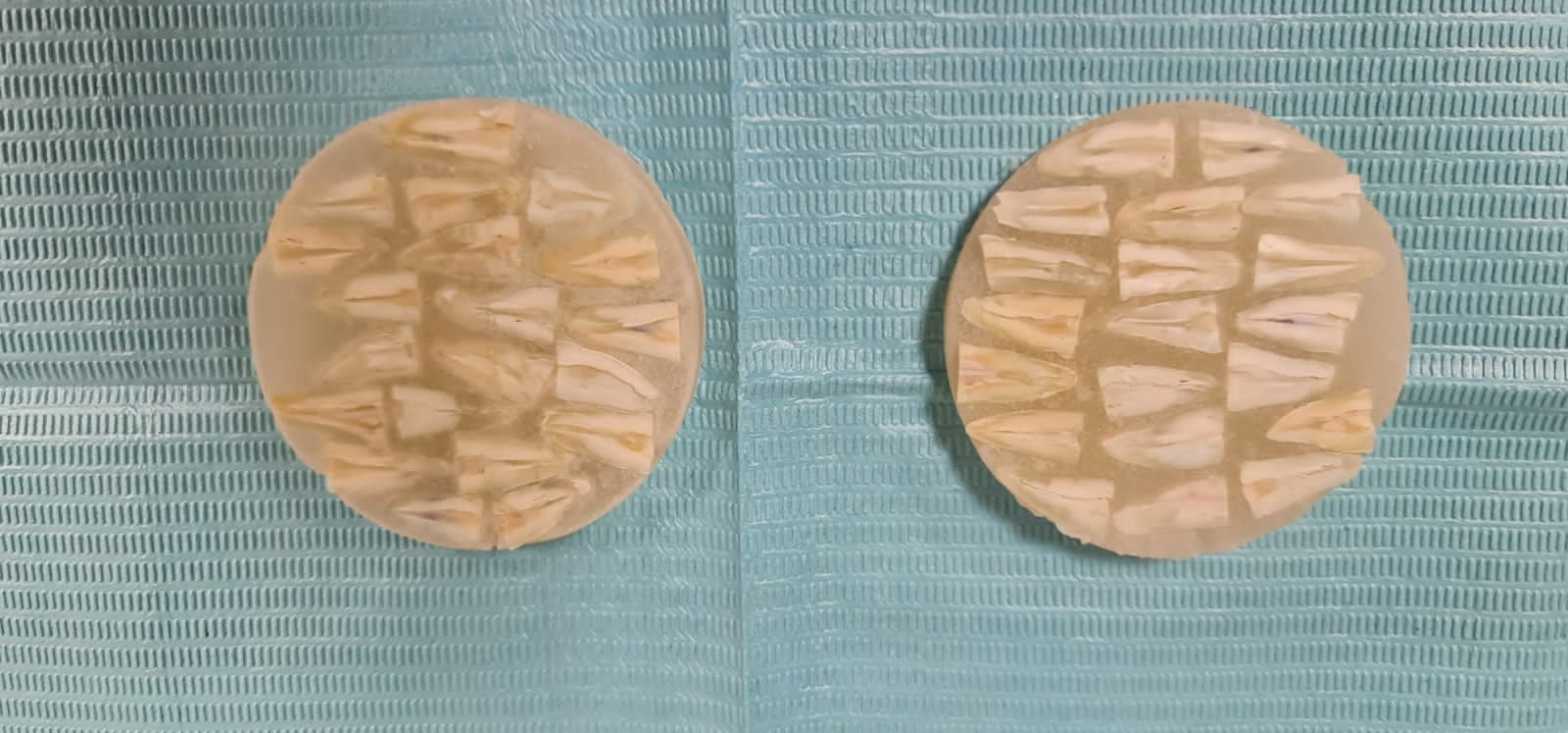
Samples embedded in autopolymerizing resin.

**Fig 2:**
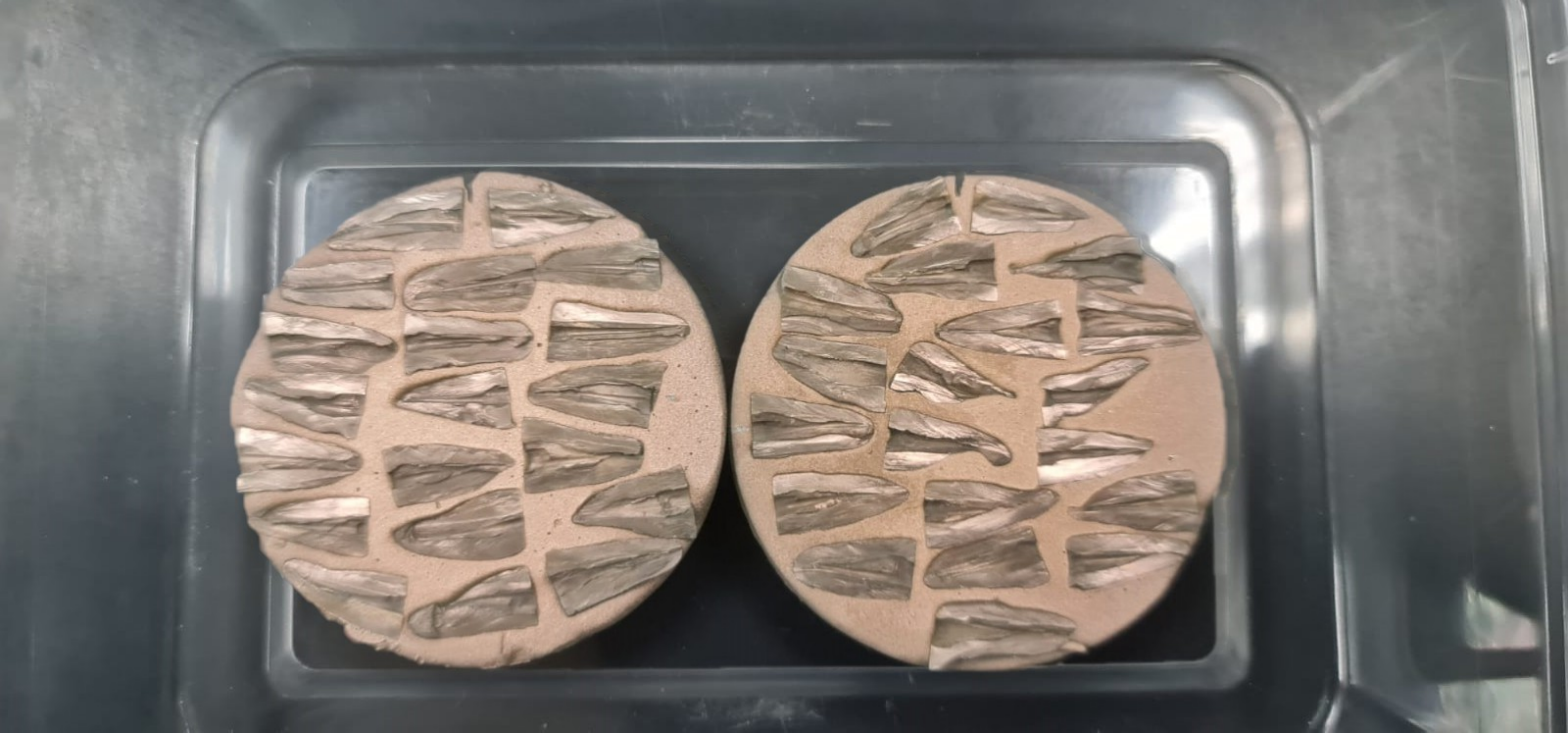
Samples sputter-coated with a gold layer.

Two longitudinal grooves were prepared on the buccal and lingual surfaces of each root using a fine 0.5-mm diamond disc (Kerr, USA), avoiding penetration into the root canals. Subsequently, the roots were split into two halves using a chisel and a hammer (HuFriedy, USA). One randomly chosen half of each root was embedded in autopolymerizing resin, then fixed in 2% glutaraldehyde and dehydrated with ethyl alcohol (Sigma Aldrich, Germany) as follows: 25% for 10 minutes, 50% for 20 minutes, 75% for 20 minutes, and 100% for 30 minutes. (**Fig 1)**, sputter-coated with a layer of 20 nm gold (**Fig 2**), and mounted on the stage of a SEM for evaluation (JEOL, Tokyo, Japan), as previously described by Paqué et al. [21].

The central light of the SEM was directed at the center of the root region to initiate imaging at a 10× magnification, which was gradually increased up to 1500×. Then, the root canals were photographed at a magnification of 2,000× and a voltage of 20 kV to assess the presence of the smear layer and the amount of dentin erosion in the coronal (10–12 mm from the apex), middle (6–7 mm from the apex), and apical (1–3 mm from the apex) thirds of the root canals. Two independent evaluators, who were blind to the group allocations, conducted the scoring using the scale developed by Torabinejad et al. [22] to assess both the smear layer and dentin erosion:

- Scoring for the smear layer:
  1. No smear layer: The root canal surface is devoid of a smear layer, with all dentinal tubules clean and open.
  2. Moderate smear layer: Although the root canal surface is clear of a smear layer, the dentinal tubules contain debris.
  3. Heavy smear layer: A dense smear layer covers both the root canal surface and the dentinal tubules.
- Scoring for dentin erosion:
  1. No erosion: Dentinal tubules retain a standard appearance and size.
  2. Moderate erosion: Erosion is observed in the peritubular dentin, the denser outer layer surrounding each tubule.
  3. Severe erosion: The intertubular dentin is significantly eroded, causing the tubules to become interconnected.

### 2.3. Statistical analysis

Descriptive statistics, including the mean and standard deviation, were computed for all continuous variables. For comparisons, a one-way analysis of variance (ANOVA) test was applied, and post hoc analysis was also performed for the multiple mean differences of root canal irrigation solutions using Tukey’s honestly significant difference (HSD) test. A *p*-value of 0.05 was considered statistically significant. All data analyses were performed using the Statistical Package for the Social Sciences (SPSS) version 26 (IBM Corporation, Armonk, New York, US).

## 3. Results

### 3.1. Smear layer

Table 2 presents the overall mean comparison of the smear layer between root levels and root canal irrigation solutions. The smear layer score in the apical third for all groups was notably higher for all groups compared to the coronal and middle thirds. The only exception was the control group, which showed no significant variance across the root levels.

**Table 2:** Overall mean comparison of the smear layer between root levels and root canal irrigation solutions.

Irrigation Solutions	Coronal	Middle	Apical	*F*-test	*p*-value ^§^
	Mean ± SD	Mean ± SD	Mean ± SD		
EDTA	^a^1.00 ± 0.00	^a^1.20 ± 0.42	^b^1.80 ± 0.42	14.625	**< 0.001** **
QMix	^a^1.00 ± 0.00	^a^1.40 ± 0.52	^b^2.10 ± 0.74	11.466	**< 0.001** **
Citric acid	^a^1.10 ± 0.32	^a^1.50 ± 0.53	^b^2.20 ± 0.79	9.300	**0.001** **
Control	3.00 ± 0.00	2.90 ± 0.32	2.80 ± 0.42	1.080	0.354

§ The *p*-value was calculated using the one-way ANOVA test

** Indicates significance at *p* < 0.05.

^a,b^ Superscript differences in the same row indicate significant (*p* < 0.05) differences between groups. The post hoc test was conducted using Tukey’s HSD test.

The overall mean score of the smear layer in the control group was significantly higher than that in the other experimental groups. No significant difference was found between the groups of EDTA, QMix, and citric acid (Table 3).

**Table 3:** Overall mean comparison of the smear layer among root canal irrigation solutions.

Smear Layer	Values	*F*-test	*p*-value ^§^
	Mean ± SD		
EDTA	^a^1.33 ± 0.48	66.219	**< 0.001** **
QMix	^a^1.50 ± 0.68		
Citric acid	^a^1.37 ± 0.49		
Control	^b^2.90 ± 0.31		

^§^ The *p*-value was calculated using the one-way ANOVA test

** Indicates significance at *p* < 0.05.

^a,b^ Superscript differences in the same column indicate significant (*p* < 0.05) differences between groups. The post hoc test was conducted using Tukey’s HSD test.

### 3.2. Dentin erosion

Table 4 presents the overall mean comparison of dentin erosion between irrigation solution groups across different root levels; no significant differences were observed.

**Table 4:** Overall mean comparison between levels of dentin erosion and root canal irrigation solutions.

Irrigation Solutions	Coronal	Middle	Apical	*F*-test	*p*-value ^§^
	Mean ± SD	Mean ± SD	Mean ± SD		
EDTA	1.50 ± 0.53	1.80 ± 0.42	2.10 ± 0.74	2.700	0.085
QMix	1.60 ± 0.52	1.40 ± 0.52	1.30 ± 0.48	0.913	0.413
Citric acid	2.40 ± 0.69	2.10 ± 0.57	2.30 ± 0.67	0.553	0.582

§ The *p*-value was calculated using the one-way ANOVA test.

When assessing the overall mean comparison of dentin erosion among root canal irrigation groups, a significant difference emerged (*F* = 15.160; *p* < 0.001). The citric acid group exhibited the highest erosion, followed subsequently by the EDTA and QMix groups (Table 5).

**Table 5:** Overall mean comparison of dentin erosion among root canal irrigation groups.

Dentin erosion	Values	*F*-test	*p*-value ^§^
	Mean ± SD		
EDTA	^a^1.80 ± 0.61	15.160	**< 0.001** **
QMix	^b^1.43 ± 0.50		
Citric acid	^c^2.27 ± 0.64		

§ The *p*-value was calculated using the one-way ANOVA test

** Indicates significance at *p* < 0.05.

^a,b,c^ Superscript differences in the same column indicate significant (*p* < 0.05) differences between groups. The post hoc test was conducted using Tukey’s HSD test.

**Fig 3:**
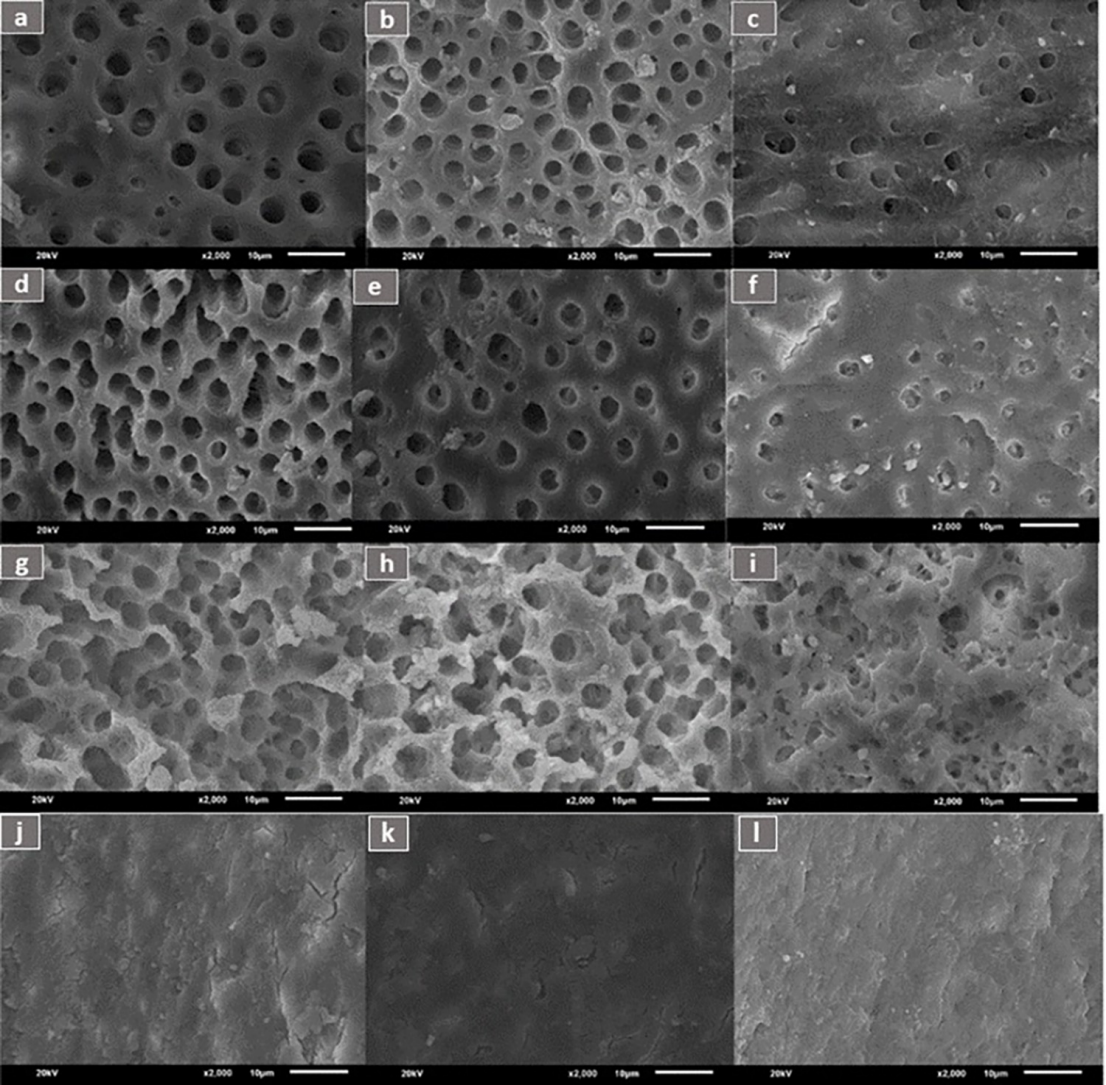
Representative SEM images of root canal wall dentin after final irrigation with different irrigants under 2,000× magnification. EDTA (a) coronal third, (b) middle third, and (c) apical third. QMix (d) coronal third, (e) middle third, and (f) apical third. Citric acid (g) coronal third, (h) middle third, and (i) apical third. Normal saline (j) coronal third, (k) middle third, and (l) apical third.

Fig 3 depicts SEM scans of samples from various experimental groups.

## 4. Discussion

Chemomechanical preparation plays a pivotal role in effectively cleaning the root canal system and removing the smear layer. Both the irrigation and instrumentation processes are deemed paramount for a successful endodontic treatment [23]. Therefore, this experimental laboratory study was designed to examine the effect of different irrigation solutions on smear layer removal and dentin erosion.

Single-rooted Mandibular premolars were selected due to their high incidence of single roots, canals, and foramina. Their ready availability, stemming from frequent extractions for orthodontic purposes, further made them a suitable choice.

In this investigation, a nickel-titanium rotary ProTaper Next system was employed. Its appeal lies in its gradually shifting tapers along the length of the cutting blades, allowing for swifter instrumentation compared to some manual techniques and fewer recapitulations, particularly in narrower or more curved canals [24]. For irrigants to reach the apical third, the minimum instrumentation size #30 (ISO) is required [25]. Therefore, all the samples were instrumented using the X3 file system of ProTaper Next, which corresponds to ISO #30.

The choice of using normal saline as a control is underpinned by its pH, which closely mirrors that of saliva. The selection of 17% EDTA and 40% citric acid as irrigation and chelating agents stemmed from their extensive application in endodontic treatments. QMix was chosen for this study as it represents a newer irrigation and chelating agent known for its dual action on both organic and inorganic components of debris [26, 27]. Moreover, a recent study showed that QMix had a similar effect to NaOCl in periapical repair [28].

As the principal irrigant between each instrument, 5.25% sodium hypochlorite was used in the current chemomechanical preparation research, it is successful in removing the smear layer’s organic material.

This study standardized both the irrigation volume (5 ml) and the duration (2 min). After following instrumentation and irrigation protocols, the samples were split longitudinally using a chisel and a hammer. As a consequence, some samples were destroyed during the sectioning phase of the experiment. While sectioning with an electric saw blade might have been more efficient and convenient, it could introduce debris and potentially affect the results. Therefore, a chisel and hammer were chosen.

SEM evaluation is considered a reliable tool for assessing smear layer removal and dentin erosion on root canal walls after endodontic preparation. Although other visualization methods, such as light microscopy, stereomicroscopy, and absorption spectrophotometry, can be used for similar purposes, qualitative analysis via SEM allows for even higher magnifications, exceeding 10,000× [29].

Regarding the smear layer, a notable difference existed between the control group and the other groups tested. However, there were no significant differences between EDTA, QMix, and citric acid. Similarly, the studies of Mankeliya et al [30] and Takada et al [31] found no significant difference between EDTA and citric acid in smear layer removal.

In terms of dentin erosion, citric acid produced significantly more dentin erosion than the other tested groups. Thus, the null hypothesis was rejected.

The control group was excluded from the erosion assessment due to the presence of a heavy smear layer that coats the dentinal tubules, making an accurate assessment of dentin erosion unfeasible. A similar approach was adopted in the study by Akçay et al. [32].

In this study, the visual analysis of the smear layer’s presence utilized Torabinejad’s scoring system [22]. This simple scoring system enhances both the validity and reliability of the study [33].

The findings of this study are consistent with others [22, 34, 35], where the highest mean score of smear layer presence was observed in the apical third, irrespective of the irrigation method used.

The removal of the smear layer in the coronal and middle thirds tends to be easier due to greater accessibility and to the larger canal diameter in the coronal and middle third, this exposes the dentin to a higher volume of irrigants, allowing a better flow of the solution.

Moreover, the apical region presents complexities, including variations in curvature, canal size, taper, diameter, ramifications, deltas, isthmuses, and dentin permeability [36]. Such findings support the use of chelating solutions, particularly in the coronal and middle thirds of the root canals. Conversely, the constricted nature of the apical third can impede the flow of irrigating and chelating solutions, potentially compromising smear layer removal [37]. Additionally, dentin sclerosis in the apical third might affect the smear layer removal and make it more challenging. [38]

The presence of the smear layer was significantly higher in the control group than in the experimental group. This could be attributed to the use of normal saline, which is ineffective in demineralizing the organic and inorganic contents of the smear layer. This result is consistent with several published studies [39–41], all indicating no effect of normal saline on smear removal.

None of the groups demonstrated complete removal of the smear layer from the root canal. This is in line with a previous study by Darrag [42], who confirmed that neither 17% EDTA nor 10% citric acid could completely remove the smear layer, especially in the apical root region of the root. The findings are also in agreement with the results of Patel et al. [43], who found that EDTA was more effective in smear removal compared to other solutions, as well as those of Vlad et al. [44], who demonstrated that 17% EDTA was more efficient than 10% citric acid in smear removal. Notably, past studies have not explored the use of 40% citric acid. While one investigation utilized 20% citric acid, it concluded that 17% EDTA was significantly more effective than the 20% citric acid solution [45]. The use of 40% citric acid in the current study did not prove to be efficient compared to the 17% EDTA solution. The efficacy of 17% EDTA can be linked to the production of soluble calcium chelates when calcium ions and EDTA interact during irrigation. Additionally, EDTA can demineralize dentin up to a depth of 20 to 30 μm within 5 min [46]. Interestingly, a recent study demonstrated that NaOCl without EDTA can efficiently remove the organic and collagen debris from the root canal surface with laser agitation [34]. Nd: YAG laser and EDTA 17% demonstrated satisfactory smear layer removal properties from the canal, significantly higher than 5.25% NaOCl and 17% EDTA [47].

In contrast, this study contradicts the findings of Stojicic et al. [15], who determined that QMix and EDTA solutions had equivalent smear removal efficiencies. Although this study noted no substantial disparity between QMix and the EDTA solution, a pronounced efficacy difference was apparent between them.

Dentin erosion refers to the chemical process wherein mineralized tooth material is lost due to exposure to acids. The pronounced dentin erosion observed in the citric acid group could be attributed to citric acid’s decalcifying nature, particularly since a high concentration (40%) was employed in this study [48].

In prior research, a 19% citric acid concentration was noted to widen the superficial portions of the dentinal tubules [49]. The findings are in line with those of Baldasso et al. [50], who found increased dentin erosion with the use of citric acid. A similar, albeit milder, adverse effect was observed when a 17% EDTA solution was employed. According to previous research, as exposure times increased, erosion increased significantly. Hence, it has been recommended to restrict the use of 17% EDTA to no more than 1 min [51]. On the contrary, another study found similar amounts of erosion in dentin in 1 and 3 minutes of applications for EDTA, Glycolic acid (GA), and Etidronic acid (HEDP) solutions with sonic activation [52].

Such observations align with earlier research, indicating that erosion of both peritubular and intertubular dentin can be attributed to these chelating agents [53]. In the present study, a notably higher concentration of citric acid, 40%, was utilized. Interestingly, both of 10% citric acid and 40% citric acid declined microhardness of root canal dentin similarly without a significant difference [54].

In contrast, the lower erosion score observed in the QMix group may hint at its relatively gentler nature. An overall score of 1.43 ± 0.50, in contrast to scores of 1.80 ± 0.61 and 2.27 ± 0.64 for EDTA and citric acid, respectively, might suggest that QMix has a smaller effect on peritubular or intertubular erosion, despite the fact that it contains EDTA, CHX, and a detergent as a surfactant. The inclusion of the surfactant likely ensures that QMix’s efficacy in smear layer removal parallels that of the EDTA solution without adversely affecting dentin erosion [8]. This is in line with the outcomes of a previous study, which postulated that the Ca/P ratio in root canal dentin remains largely unaltered when treated with Qmix [55]. In addition, QMix is less decalcifying and erosive than 17% EDTA [8]. However, these results challenge earlier findings, which posited that QMix had no tangible impact on dentin erosion [50]. The use of a combination of NaOCl and HybenX, efficiently removes smear layer and produces a lower degree of erosion if compared with 17% EDTA [56].

Erosion undeniably imposes detrimental changes on dentin, affecting its surface roughness, microhardness, and nanohardness, which can compromise the tooth’s fracture resistance. As such, previous research underscores the cautious use of irrigation agents. In line with this, Vollenweider et al. [57] detailed that after 2 h of exposure, 17% EDTA reduced the fracture resistance and elastic modulus by one-third and half, respectively.

As discussed above, several studies compared the effect of different root canal irrigants on smear layer removal and dentin erosion, however this study is novel as it is the only published work that studied the effectiveness of initial irrigation with sodium hypochlorite (NaOCl) and final irrigation with QMix, 40% citric acid, and 17% ethylenediaminetetraacetic acid (EDTA) in particular on smear layer removal and dentin erosion using Scanning Electron Microscope.

One limitation of this study was that the experiments were performed at room temperature instead of body temperature. Another limitation was that the evaluation of the erosion degree was carried out only through a surface qualitative score. It would have been better to evaluate the effects in terms of micro- or nanohardness and mineral content.

Further studies are needed to examine the effect of different irrigant activation methods and needle gauges on smear layer removal and dentin erosion. The effect of irrigation solutions on the push-out bond strength of cements with root dentin could be investigated in detail. Moreover, quantitative analysis using plasma-atomic emission spectrometry is also advised for determining the percentage weight of mineral contents of root canal dentin after irrigation protocols.

## 5. Conclusions

The smear layer removal was significantly more effective in the coronal and middle thirds than in the apical third, irrespective of the group. Normal saline exhibited the lowest efficacy in smear layer removal. However, no significant differences were observed between EDTA, QMix, and citric acid. Regarding dentin erosion, there were notable differences among the tested groups: 40% citric acid led to significantly greater dentin erosion, followed by 17% EDTA and QMix. Hence; EDTA, QMix, and citric acid are capable irrigants to remove smear layer from all thirds of the canals. However, 40% citric acid is not advocated as it produced significant dentin erosion, which might weaken the tooth structure.

## Supporting information

S1 Dataset (XLSX)
